# Transcriptome analysis identifies a robust gene expression program in the mouse intestinal epithelium on aging

**DOI:** 10.1038/s41598-019-46966-3

**Published:** 2019-07-18

**Authors:** Juri Kazakevych, Elena Stoyanova, Anke Liebert, Patrick Varga-Weisz

**Affiliations:** 1 Babraham Institute, Cambridge, CB22 3AT UK; 2 Genomics and Computational Biology, School of Biological Sciences, University of Essex, Colchester, UK; 3 Present Address: The Francis Crick Institute, London, NW1 1AT UK

**Keywords:** Endoderm, Intestinal stem cells, Stem-cell niche, Transcriptomics, Ageing

## Abstract

The intestinal epithelium undergoes constant regeneration driven by intestinal stem cells. How old age affects the transcriptome in this highly dynamic tissue is an important, but poorly explored question. Using transcriptomics on sorted intestinal stem cells and adult enterocytes, we identified candidate genes, which change expression on aging. Further validation of these on intestinal epithelium of multiple middle-aged versus old-aged mice highlighted the consistent up-regulation of the expression of the gene encoding chemokine receptor Ccr2, a mediator of inflammation and several disease processes. We observed also increased expression of *Strc*, coding for stereocilin, and dramatically decreased expression of *Rps4l*, coding for a ribosome subunit. *Ccr2* and *Rps4l* are located close to the telomeric regions of chromosome 9 and 6, respectively. As only few genes were differentially expressed and we did not observe significant protein level changes of identified ageing markers, our analysis highlights the overall robustness of murine intestinal epithelium gene expression to old age.

## Introduction

Despite the fact that the intestinal tract is critical for life and health, there is limited knowledge on how intestinal health is maintained throughout lifespan. Many age-related intestinal disorders, such as colorectal cancer, diverticular disease and inflammatory bowel disease (IBD) have become common in developed countries with changes in lifestyle, particularly nutrition, potentially underlying these pathologies. Aging affects intestinal function and health^1^. Age is also a strong risk factor for colon cancer, one of the most common forms of cancer. Gastrointestinal conditions are some of the most common reasons for morbidity and hospitalization of the aged in Europe and are still on the rise^2^.

Interestingly, in various model organisms, such as *Caenorhabditis elegans* and *Drosophila*, the intestine is the most important organ in determining lifespan and health^3,4^. In humans, aging is linked to increased intestinal permeability and innate immunity responses^5,6^.

Aging, even ‘healthy’ aging is associated with changes in gene expression, including in the gut^5–7^. A study that compared intestinal stem cells (ISC) from young (2–4 months) and old (18–22 months old) mice showed that aging is associated with an increase in ISC number, proliferation and an increase in ISC apoptosis^8^. However, little is known about how and to what extent aging affects gene expression in intestinal stem cells, which drive the constant and essential regeneration of the intestinal epithelium. Our own and other works, including single cell transcriptomic analysis, suggest gene expression changes in somatic stem and precursor cells of the hematopoietic system upon aging^9,10^. Thus, it is likely that these also happen in mammalian intestinal stem cells and their derivative cell types. It is, therefore, important to understand the effects of aging on intestinal cell gene expression and function. Here we performed a transcriptome-based screen to identify genes that show expression changes in old age in cells from the murine intestinal epithelium. We identified *Ccr2* and *Strc* upregulation and *Rps4l* downregulation as a hallmark for aged intestinal cells on the background of an overall robust gene expression program.

## Results

### RNAseq identifies candidate age-regulated genes in intestinal epithelium cells

We used Lgr5‐EGFP‐IRES‐CreERT2 knock‐in mice to isolate *Lgr5*+ intestinal stem cells (ISC)^11,12^ and adult enterocytes (AE). *Lgr5*-EGFP^high^ cells and AE were isolated from younger (~33 weeks) and old mice (~118 weeks) via fluorescence-activated cell sorting using published procedures^12,13^ (Fig. S1). Choosing mice that are middle-aged for the younger cohort allows the investigation of the aging process un-obscured by changes occurring during early adulthood development. Poly(A)+ RNA was isolated from both cell populations and gene expression was analyzed by RNA sequencing (RNAseq). This showed that the sorted ISC expressed known stem cell markers, such as *Olfm4* and *Lgr5*^12^, validating the cell purification scheme (Figs S2 and S3B). Similarly, AE expressed known differentiated enterocyte markers *Vil1*, *Apoc3* and *Sis*^13,14^. The expression pattern of proliferation (e.g., *Myc*) and cell type markers (e.g., *Lgr5*, *Vil1*) did not change with aging, indicating their robust expression (Figs S2 and S3B).

**Figure 1:**
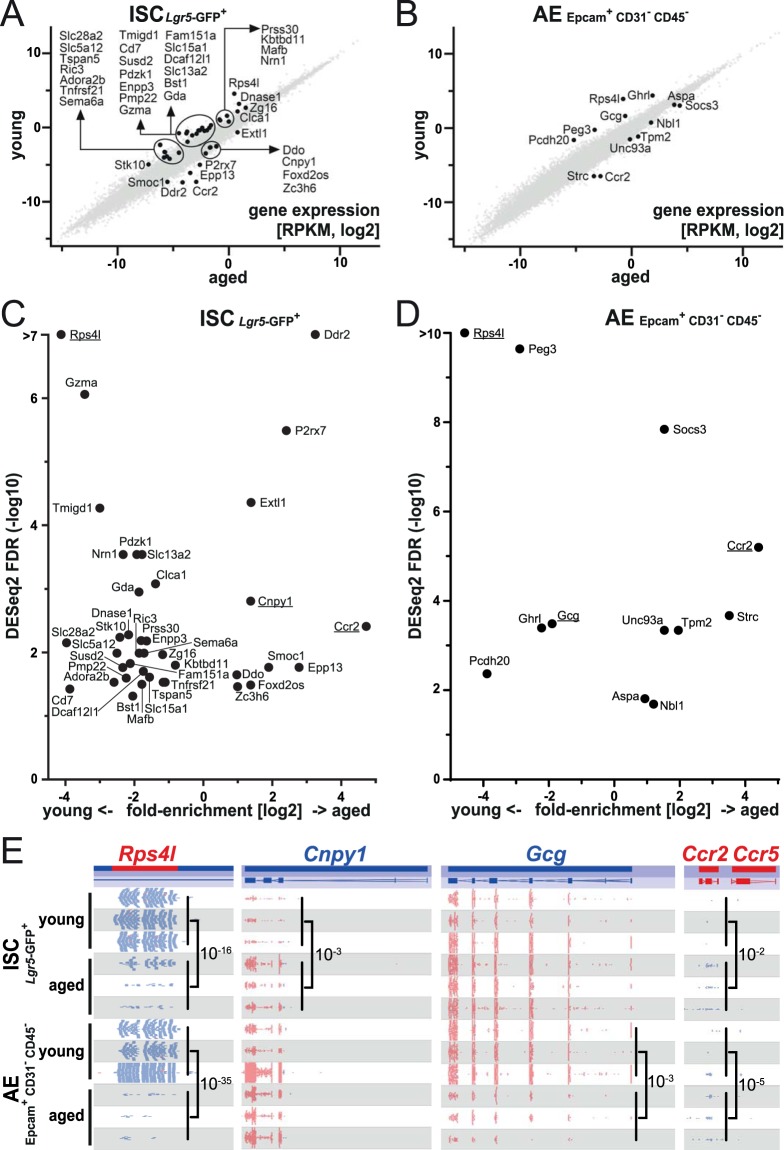
Gene expression changes upon aging in small intestinal stem cells (ISC) and adult enterocytes (AE). (**A**,**B**) Scatterplots show differentially regulated genes in ISC (**A**) and AE (**B**) and provide information regarding their relative expression levels as log2 scale RPKM quantitation. All genes shown and significantly up/down-regulated genes (DESeq2 test with cut-off FDR < 0.05, n = 3) are highlighted. (**C**,**D**) the same data as in (**A**,**B**) shown as Volcano plots, depicting significantly up/down-regulated genes (DESeq2 test with cut-off FDR < 0.05, n = 3) in ISC (**C**) and AE (**D**). X-axis: Old/Young log(2) fold-enrichment of reads per transcript. Y-axis: -log(10) DESeq2 FDR. Genes shown in **E** are underlined. High FDR values are capped at indicated maximum Y-values for visualization. (**E**) RNAseq track examples of genes differentially expressed between young and aged ISC and AE samples. *Rps4l* (0.94 kb), *Cnpy1* (45 kb), *Gcg* (9.1 kb) and *Ccr2* (12 kb). Significant old/young changes in gene expression identified by DESeq2 are indicated with corresponding FDR values. Normalized quantitation is shown in Fig. S3A.

We identified 10 significantly age-upregulated genes and 29 age-downregulated genes in stem cells, as well as 7 age-upregulated and 5 age-downregulated genes in adult enterocytes (Fig. 1). Gene ontology (GO) analysis of the combined 49 gene list identified among others the following terms: inflammatory response (P = 0.026), myeloid cell activation involved in immune response (P = 0.018), negative regulation of response to external stimulus (P = 0.031) and intrinsic component of plasma membrane (P = 0.0004) (for analysis and result details see Supplementary Table 1, Fig. S4). Notably, *Ccr2*, the most strongly up-regulated differentially expressed gene (DEG) in aged mice, was found in both the ISC and AE cells, as was the most down-regulated DEG, *Rps4l* (Fig. 1).

### Increased expression of *Ccr2* and *Strc* and decreased expression of *Rps4l* mRNAs are markers of aged intestinal epithelial cells

As aging is a heterogeneous process, with even isogenic individuals succumbing to age-associated changes at different rates, we validated candidate genes from the RNAseq data by reverse transcription followed by quantitative polymerase chain reaction (RT-qPCR) using cells extracted from individual mice of a larger additional cohort of aged (~109 weeks) and young mice (~37 weeks). For the validation of our RNAseq results, we isolated crypts, as these contain mostly ISC and cells derived directly from ISC: transit amplifying (TA) cells and Paneth cells. The more abundant TA cells emerge from ISCs at the beginning of intestinal differentiation with an expression pattern similar to ISC. Therefore, we did not expect substantial effects on the observed age-related changes in gene expression. AE were isolated from villi as was done for RNAseq.

**Figure 2:**
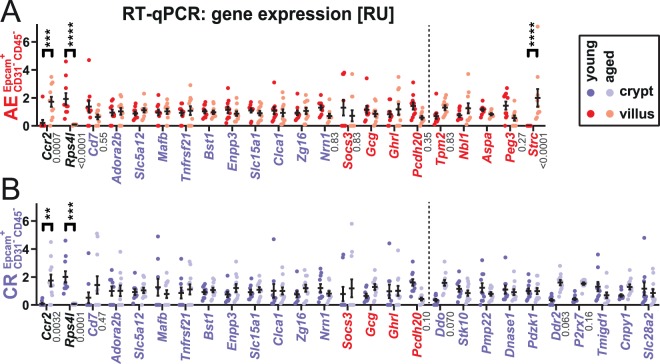
Validation of expression changes on aging in selected genes by RT-qPCR. AE (**A**) and Crypt epithelium (CR) (**B**) mRNA levels were normalized to *Actg1* and cell type-specific target gene signal average on a linear scale. Genes differentially expressed between young and aged animals are indicated, significance threshold P < 0.05 (2-way ANOVA by cell type, Holm-Sidak multiple testing correction, n = 9–11, **P < 0.01, ***P < 0.001, ****P < 0.0001, all P-values < 0.9 are indicated). Genes initially identified as DEGs in the ISC RNAseq are in purple letters, those identified in the AE RNAseq in red and those identified in both in black. Genes right of the dashed line were tested only in a single cell type.

On the AE samples we tested 11 out of the 12 DEGs identified in the AE RNAseq (for technical reasons, we could not test *Unc93a*) and further 11 genes that were identified in the ISC RNAseq (selected, because these genes were significant DEGs in the latter list and some are linked to inflammatory responses, Fig. 2A). On the small intestinal crypt samples, we tested 23 of the 39 candidate genes that were identified in ISC, as well as 4 additional genes that were identified in AE but did not show significant changes in the ISC RNAseq (Fig. 2B). Of the 32 tested genes, only 3 genes were significantly differentially regulated between young and aged. In both crypts and AE, *Ccr2* was up-regulated on aging, while *Rps4l* was markedly down-regulated. Furthermore, in AE we validated *Strc* as being significantly upregulated on aging. The expression of three other genes, *Ddo*, *Ddr2*, and *P2rx7* was also increased on average in crypts, and *Pcdh20* was decreased in crypts and AE, but these changes missed the significance threshold of P < 0.05.

To test if changes in gene expression lead to changes in protein levels on aging, we performed Western blot analysis on crypts and villi extracts (Fig. S5). We selected targets where we observed the strongest transcript level differences and where suitable antibodies were available. For Ccr2 and Ddo we detected no changes in protein levels. For Ddr2 and Strc the antibodies identified proteins of various sizes and in the case of Ddr2 there is some increased signal observed on aging in the crypts fraction.

**Figure 3:**
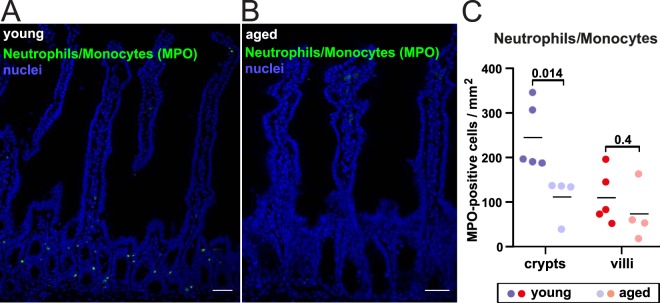
Neutrophils and monocytes in the small intestinal epithelium during ageing. (**A**,**B**) Intestinal epithelium localization of Myeloperoxidase (MPO)-positive cells (green) by immunofluorescence staining and nuclear counterstaining with DAPI (blue) shown as maximum intensity projections of young (**A**) and aged (**B**) small intestinal epithelium (SI). Scale bars: 40 µm. Increased magnification of crypt compartments in (**A**,**B**), as well as negative staining controls are shown in Fig. S6. (**C**) Quantification of F4/80-positive cells per imaged crypt/villus area of the young and aged SI. Indicated P-values determined by 2-way ANOVA with Holm-Sidak’s multiple comparisons test (n = 4–5). Raw quantification data and full statistical results are shown in Supplementary Table 4. Scoring examples are shown in Fig. S6A,C.

As Ccr2 is involved in monocyte and neutrophil recruitment^15,16^, we stained small intestine epithelium sections with an antibody against myeloperoxidase, which detects neutrophils and monocytes, to monitor if there is changed recruitment of these cells in aged compared to younger tissue. We found the number of MPO positive cells drops in the aged tissue (Fig. 3 and Supplementary Fig. S6).

## Discussion

RNAseq followed by RT-qPCR analysis of the identified candidate genes indicated that only a handful of genes are consistently differentially expressed in the intestinal epithelial cells between young and aged animals. This may reflect the highly robust gene expression machinery in this dynamic tissue. It also highlights the excellent health status of the aged mice colony at the Babraham Institute, as we have little evidence of upregulation of markers linked to inflammation, with the notable exception of *Ccr2* and *P2rx*7.

*Ccr2* encodes a chemokine receptor that binds to Ccl2 (also called Mcp-1 for Monocyte Chemoattractant Protein 1). The Ccl2/Ccr2 axis mediates inflammatory and innate immunity processes by recruiting monocytes and neutrophils to inflammation sites^15^ (reviewed in^16^) and has been implicated in several pathological processes, including metastasis, Alzheimer’s disease, autoimmune disease, atherosclerosis and obesity^16^. Relevant to the context of this study, CCR2 has been shown to be increased in patients with inflammatory bowel disease and plays a critical role in inflammatory processes in experimental colitis in mice^17–21^. Inhibition of Ccl2 or Ccr2 mediated signaling prevented disease progression in mouse models of colitis^22–24^. We found that *Ccl2* is completely silenced in the intestinal epithelial cells (Fig. 3B). There is little known about the direct role of Ccr2 in intestinal epithelial cells in these processes. Ccr2 is involved in age-related macular degeneration as well as aging T-cells^25–27^. Previous studies have shown that aging is linked to increased expression of markers of inflammation in various tissues (often referred to as ‘inflamm-aging’, reviewed in^28^). Age related inflammation has so far not been linked to Ccr2 in the intestine. As we did not find evidence for increased protein levels of Ccr2 in the intestinal epithelium in steady-state conditions nor an increased neutrophil/monocyte (MPO^+^ cells) recruitment to the epithelium upon aging, the physiological implication of the increased *Ccr2* mRNA is unclear. If anything, we found a drop in the numbers of MPO positive cells, potentially indicating a changed immune response, similar to what has been published before^29^. It is striking that there is a discrepancy between the changing gene expression of *Ccr2*, while protein levels do not seem affected. This might be due to mechanisms affecting protein translation or stability that buffer Ccr2 protein levels.

We found the mRNA expression of *Strc* to be increased in the aged intestinal epithelium. Its encoded protein stereocilin is involved in cochlear function^30^, but to our knowledge nothing is known about an intestinal role. Very little is known regarding the role of *Rps4l*, which potentially encodes a pseudogene. It is notable that of the 3 DEGs that we could validate in our RT-qPCR approach, *Ccr2* and *Rps4l* are expressed from genes situated at telomeric regions: *Ccr2* at the telomere of chromosome 9 (distance to unmappable telomeres: 0.5 Mbp) and *Rps4l* close to the telomeric region of chromosome 6 (distance to unmappable telomeres: 1 Mbp). In humans, aging has been shown to be linked to telomere shortening in the intestinal tissue^31,32^. This, in turn, may lead to expression changes. Whether alterations of telomere structure lead to the observed changes in gene expression in the intestinal epithelium of the aged mice will require further investigation. Gene expression changes between otherwise isogenic strains might be due to genetic drift. However, the fact that we observed the changes in *Ccr2*, *Strc* and *Rps4l* in two completely different cohorts (the Lgr5‐EGFP‐IRES‐CreERT2 knock‐in mice and the aged-mice colony of Babraham Institute) suggests that this does not explain the observations.

The other genes that show a trend of expression changes on aging in cells from the small intestinal crypt fraction are noteworthy. *Ddr2* codes for a transmembrane collagen receptor with tyrosine kinase activity and has been implicated in various disease processes, such as atherosclerosis and cancer^33^. *Ddo* codes for a D-aspartate oxidase, the promoter of which has been shown to lose DNA methylation on aging in humans in several studies^34–36^. *Pcdh20* codes for a protocadherin, a homophilic cell-adhesion protein, and has been suggested to function as a tumour-suppressor gene^37,38^. The role of PCDH20 in the intestinal epithelium is poorly understood. *P2rx7* codes for a purinergic receptor, a mediator of inflammation and inflammatory pain through its regulation of IL-1β processing and release, including in the gut^39^.

Our study shows marked differences from previous studies. Moorfield *et al*.^8^ examined expression changes among 96 candidate genes between young and old ISC and adult enterocytes from mouse with quantitative RT-PCR and identified several changes that we did not. We note substantial differences in our approaches, notably Moorfield *et al*. compared younger (2–4 months) versus older (18–22 months) mice, while we compared middle aged mice (~8 months) versus old mice (~27 months), hence investigating processes specific to late aging.

In a human study, age-linked changes in intestinal permeability have been associated with up-regulation of claudin-2 mRNA expression, albeit there were no changes in claudin-2 protein distribution^5^. We did not observe changes in claudin gene expression in our analyses. As mice and humans have different life spans, nutrition, microbiomes and genome structures, the effect of aging on gene expression of human intestinal epithelium needs further exploration.

In summary, our data underscore the robustness of the gene expression program with few transcriptional changes and evidence for tight post-transcriptional control in the aging gut. Although the epithelial transcriptome is very resilient to aging, age-related changes occur to the overall digestive system, as was previously shown for the microbiome composition^29,40–43^ and components of the immune system (reviewed in^44^, this study).

## Methods

### Mice

Male mice with C57BL/6 background were used for all experiments. The healthy aged colony of C57BL/6 male mice was established under Specific Pathogen Free (SPF) conditions and were maintained in accordance with local and Home Office rules and ARRIVE guidelines. All mice were fed *ad lib*. The animals for RNAseq were on average 118 weeks old in the aged cohort (115,119 and 122) and 33 weeks in the young cohort (31,33 and 35). For RT-qPCR the old cohort averaged at 109 weeks (105–118, 11 mice) and the young cohort at 37 weeks (25–43, 10 mice). Mice in RNAseq cohorts were *Lgr5*-GFP positive to facilitate ISC-isolation (Lgr5-EGFP-IRES-creERT2 mice, obtained from Jackson Laboratory). Experimental protocols were approved by the Babraham Research Campus local ethical review committee and the Home Office (PPL 70/8994 and 80/2529).

### Cell extraction from small intestinal crypts and villi

Mice were killed by exposure to CO_2_ followed by cervical dislocation. Small intestines were extracted, opened longitudinally and washed in phosphate buffered saline (PBS) followed by isolation of crypt and villus fractions at room temperature as described^13^. For obtaining single cell suspensions from of intestinal crypts, we used Dispase II (Sigma D4693, 0.05 mg/ml final concentration), alongside DNase (Qiagen 79254, 20 Kunitz U/ml final concentration) and Collagenase (Sigma C7657, 0.15 mg/ml final concentration).

### Isolation of RNA from AE, ISC and whole crypt epithelium

Villus cell suspensions for RNAseq and RT-qPCR as well as crypt cell suspensions for RT-qPCR were stained in 2% fetal bovine serum (FBS)/PBS with 1:200 APC-conjugated anti-Epcam antibodies (CD326, eBioscience 17-5791-82), 1:1000 AF488-conjugated anti-CD45 antibodies (Biolegend 103122) and 1:1000 AF488-conjugated anti-CD31 antibodies (Biolegend 102414) for 15 minutes at room temperature, washed 3 times and resuspended in 2% FBS/PBS with 3.2 µg/ml ROCK-inhibitor (Y-27632, Sigma). All cell preparations were stained with 4’,6-diamidino-2-phenylindole (DAPI) prior to sorting. Epcam^high^ CD31^−^ CD45^−^ DAPI^−^ cells were sorted on BD Aria III SORP cell sorter with a 100 µM nozzle (Fig. S1A,C). ISC (GFP^high^ DAPI^−^) for RNAseq were isolated from crypt suspensions of Lgr5-GFP^+^ mice and processed as above except the staining step (Fig. S1B). 60,000–100,000 cells were sorted into RLT buffer for subsequent RNA-isolation according to manufacturer’s protocol with the RNeasy Micro kit (Qiagen 74004) with on-column DNA digestion. Quantity and quality of RNA were measured with a Bioanalyzer Eukaryote Total RNA Pico assay (Agilent Technologies, 5067–1513).

### RNAseq

Libraries were generated from 35 ng total RNA according to NEB Ultra II Directional RNA Library Preparation Kit for Illumina (E7760), Poly(A) mRNA magnetic isolation module (E7490) and multiplex oligos (E7335) manuals with the following modifications: 14 PCR-amplification cycles were performed; SPRI select beads were substituted with Seramag Speedbeads (Thermo scientific 65152105050250) for size selection steps. Seramag beads were washed with TE buffer and resuspended in 50 volumes of PEG 8000 (Sigma 1546605) with 2.5 M NaCl, 10 mM Tris-Cl pH 8.0, 1 mM EDTA, 0.05% Tween 20. PEG 8000 amounts used were corresponding to 10 and 12% final PEG concentrations on sample addition. Size selection steps were performed at room temperature, adding the sample in 100 µl nuclease free water to 80 µl of bead suspension, followed by resuspension by pipetting, incubation for 10 min and precipitation on a magnetic rack. After removal of supernatant, the beads were washed twice with 80% ethanol and moderately dried before elution with TE buffer. The size selection after second strand DNA-synthesis was performed with 12% PEG concentration, the remaining size selections with 10% PEG. After PCR-amplification the size selection was performed twice (10% PEG). Quantity and quality were measured with a Bioanalyzer High Sensitivity DNA assay (Agilent Technologies, 5067–4626) and the Roche KAPA library quantification kit. Libraries were sequenced on a HiSeq. 2500 sequencer (Illumina) as HiSeq. 50 bp Single End reads according to manufacturer’s instructions.

### Bioinformatic analysis

RNAseq reads were adaptor trimmed with Trim Galore (version 0.4.4) prior to mapping to the mouse reference genome GRCm38/mm10 with HiSat2 (version 2.1.0). Uniquely mapped RNAseq data was analysed with SeqMonk version 1.42.0. Read counts were quantified using the RNAseq quantitation pipeline implemented in SeqMonk, quantifying merged transcripts over exons with 75-percentile normalization of all libraries. Differentially expressed genes were extracted from raw read count quantitation with the multiple testing corrected DESeq2 algorithm^45^ implemented in SeqMonk. GO-enrichment analysis of DEGs were performed with g:profiler^46^ against the background list of intestinally expressed genes as detailed in Supplementary Table 1.

### RT-qPCR

A subset of 32 targets was selected based on their biological significance from the 49 genes differentially expressed between young and old animals (DESeq2, FDR < 0.05) and appropriate oligonucleotide primers (Supplementary Table 2) were designed by NCBI Primer-BLAST software with murine mRNA template. Total RNA was reverse transcribed and quantitated with the Luna Universal One-Step RT-qPCR kit (NEB E3005) and BioRad thermocycler CFX384. Cq-values were per-sample normalized to *Actg1* and subsequently per-target to average target signal in each cell type. Differential expression between old and young animals was assessed for ISC and AEs separately, using 2-way ANOVA with Holm-Sidak multiple testing correction, significance threshold P < 0.05 (Graphpad Prism 7.05).

### Western blot

Small intestinal crypts and villi were extracted as described above. The epithelium was then pelleted 10 min at 500 × g, resuspended in Laemmli 2 × lysis buffer supplemented with 5% beta-mercaptoethanol and boiled for 1 min. Samples were briefly sonicated to reduce viscosity.

Antibodies against alpha-Tubulin (T9026, Sigma), Strc (Biorbyt orb313204, 1 µg/ml), Ddo (Abcam ab175110, 0.8 µg/ml), Ccr2 (ab203128, 0.66 µg/ml), Ddr2 (Abcam ab76967, 1.8 µg/ml) were used for Western blot with 3% BSA blocking, tris- buffered saline-0.1% Tween-20 (TBS-T) washing buffer and enhanced chemiluminescence (ECL) detected on x-ray film. Protein ladders were from Thermo Scientific #26616 and from GE Healthcare #RPN800E. Protein band intensity was quantified and calculated relative to their corresponding loading control signals (alpha-tubulin) after background subtraction with FIJI-software^47^ (Supplementary Table 3).

### Neutrophil/Monocyte quantitation

Immunofluorescent stainings were prepared and confocal imaging performed as described^48^ with primary Myeloperoxidase (MPO)-antibody (R&D AF3667, 5 µg/ml) incubated 2 h at room temperature and secondary anti-goat AlexaFluor 488 (Invitrogen A11055, 10 µg/ml) incubated at 4 °C over night. Permeabilization was performed for 4 min in 0.3% TritonX/PBS. 2% BSA/2% donkey serum/PBS was used as blocking solution. No unmasking was performed. Imaging was performed on a Zeiss 780 confocal microscope with a 20 × Plan Apo air objective at optimal resolution settings with 2 × line averaging. 5 × 2 µm optical stacks were processed as maximum intensity overlays (optical thickness 8 µm. Contrast enhancement (thresholding background signal) were performed with FIJI^47^. Three technical replicates (different intestinal positions within the same sample) were measured and averaged per sample. Average total imaged crypt area per sample: 0.16 mm^2^, villus area 0.18 mm^2^. Crypt and villus areas were measured with FIJI, positive cells counted manually after background correction.

## Supplementary information

Supplementary Figures S1-S6

Supplementary Table 1

Supplementary Table 2

Supplementary Table 3

Supplementary Table 4

## Data Availability

The datasets generated in this study are available in the GEO repository with accession number GSE122441.
