# Supplementary material for: Transcriptome analysis identifies a robust gene expression program in the mouse intestinal epithelium on aging
Source: Sci Rep. 2019 Jul 18;9:10410. doi: 10.1038/s41598-019-46966-3 (PMC6639340; doi:10.1038/s41598-019-46966-3)
Supplement: Supplementary file 1 — Supplementary Figures S1-S6 [file 41598_2019_46966_MOESM1_ESM.pdf]

Supplementary Figures

Transcriptome analysis identifies a robust gene expression program in the mouse intestinal epithelium on aging

Juri Kazakevych[1], Elena Stoyanova[1], Anke Liebert[1,3], Patrick Varga-Weisz[1, 2,*]

1: Babraham Institute, Cambridge CB22 3AT, UK

2: Genomics and Computational Biology, School of Biological Sciences, University of Essex, Colchester, UK

3: Current address: The Francis Crick Institute, London, NW1 1AT, UK

*correspondence: patrick.varga-weisz@essex.ac.uk

Figure S1

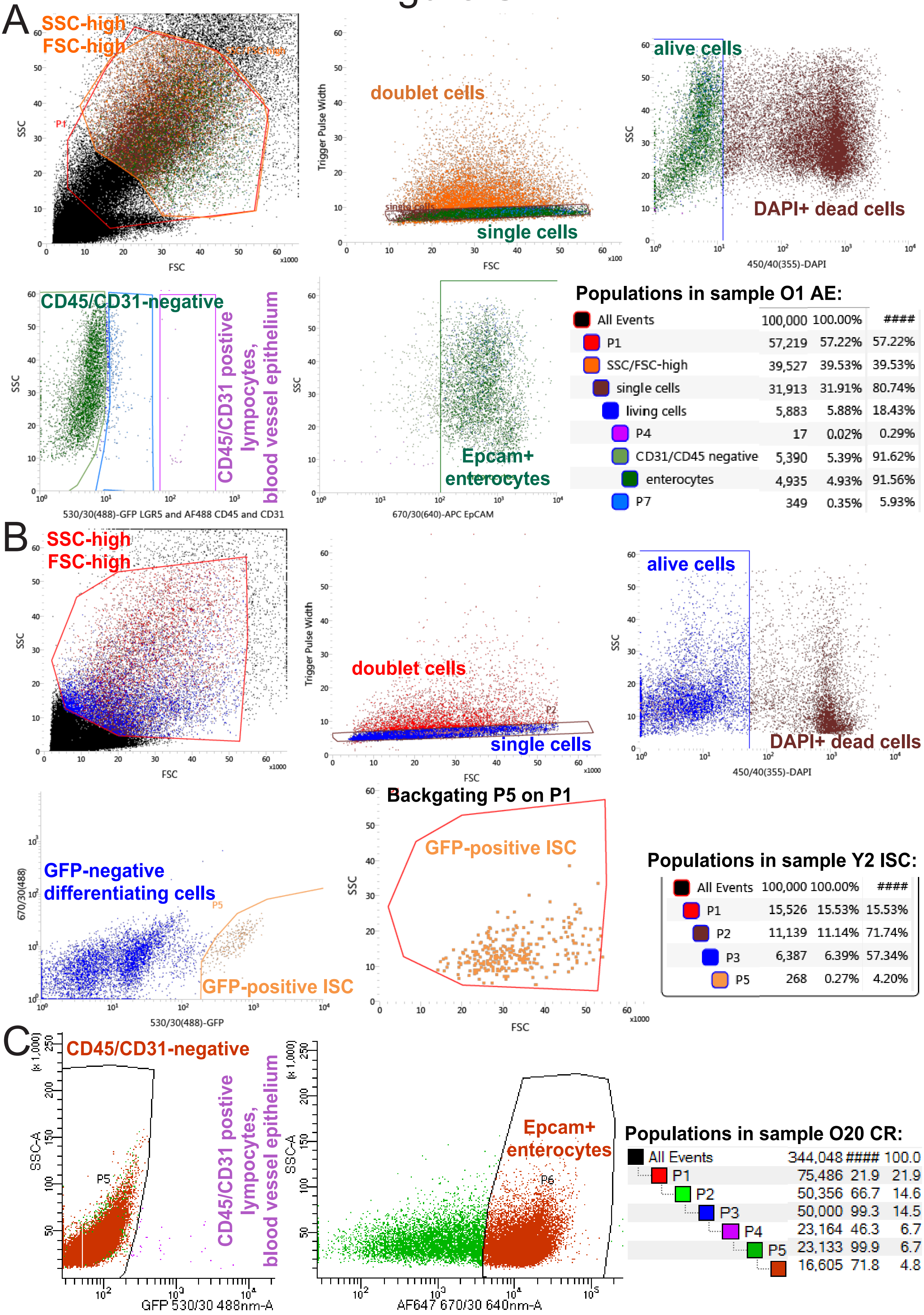

Populations in sample O1 AE:

All Events	100,000	100.00%	####
P1	57,219	57.22%	57.22%
SSC/FSC-high	39,527	39.53%	39.53%
single cells	31,913	31.91%	80.74%
living cells	5,883	5.88%	18.43%
P4	17	0.02%	0.29%
CD31/CD45 negative	5,390	5.39%	91.62%
enterocytes	4,935	4.93%	91.56%
P7	349	0.35%	5.93%

Populations in sample Y2 ISC:

All Events	100,000	100.00%	####
P1	15,526	15.53%	15.53%
P2	11,139	11.14%	71.74%
P3	6,387	6.39%	57.34%
P5	268	0.27%	4.20%

Populations in sample O20 CR:

All Events	344,048	####	100.0
P1	75,486	21.9	21.9
P2	50,356	66.7	14.6
P3	50,000	99.3	14.5
P4	23,164	46.3	6.7
P5	23,133	99.9	6.7
	16,605	71.8	4.8

Figure S1. Isolation of AE, ISC and whole crypt epithelium cells by flow sorting. Sorting strategy with exemplary samples of aged AE (**A**), young ISC (**B**) and aged whole crypt epithelium (**C**) and respective population sizes, fractions of total events and fractions of parent population. (**A**) Villus epithelium single cell suspension is gated for cell geometry via SSC (side-scatter) and FSC (forward scatter) parameters to obtain single cells and avoid cell debris. Live cells are gated for by negative DAPI staining. CD45+ and CD31+ cells (lymphocytes, blood vessel epithelium) are excluded by AF488-negative gating. AE are gated as Epcam-high cells and sorted to lysis buffer. (**B**) Crypt epithelium single cell suspension is gated until alive cells similar to **A**. *Lgr5*-GFP-positive ISC are gated as GFP-high cells and sorted to lysis buffer. Back-gating of GFP-positive ISC on the primary SSC/FSC scatter plot shows a single cell population. (**C**) Crypt epithelium single cell suspension is gated until alive cells as in **B**. CD45+ and CD31+ cells (lymphocytes, blood vessel epithelium) are excluded by AF488-negative gating. Whole crypt epithelial cells (including ISC, TA and Paneth cells) are gated as Epcam-high cells and sorted to lysis buffer.

Figure S2

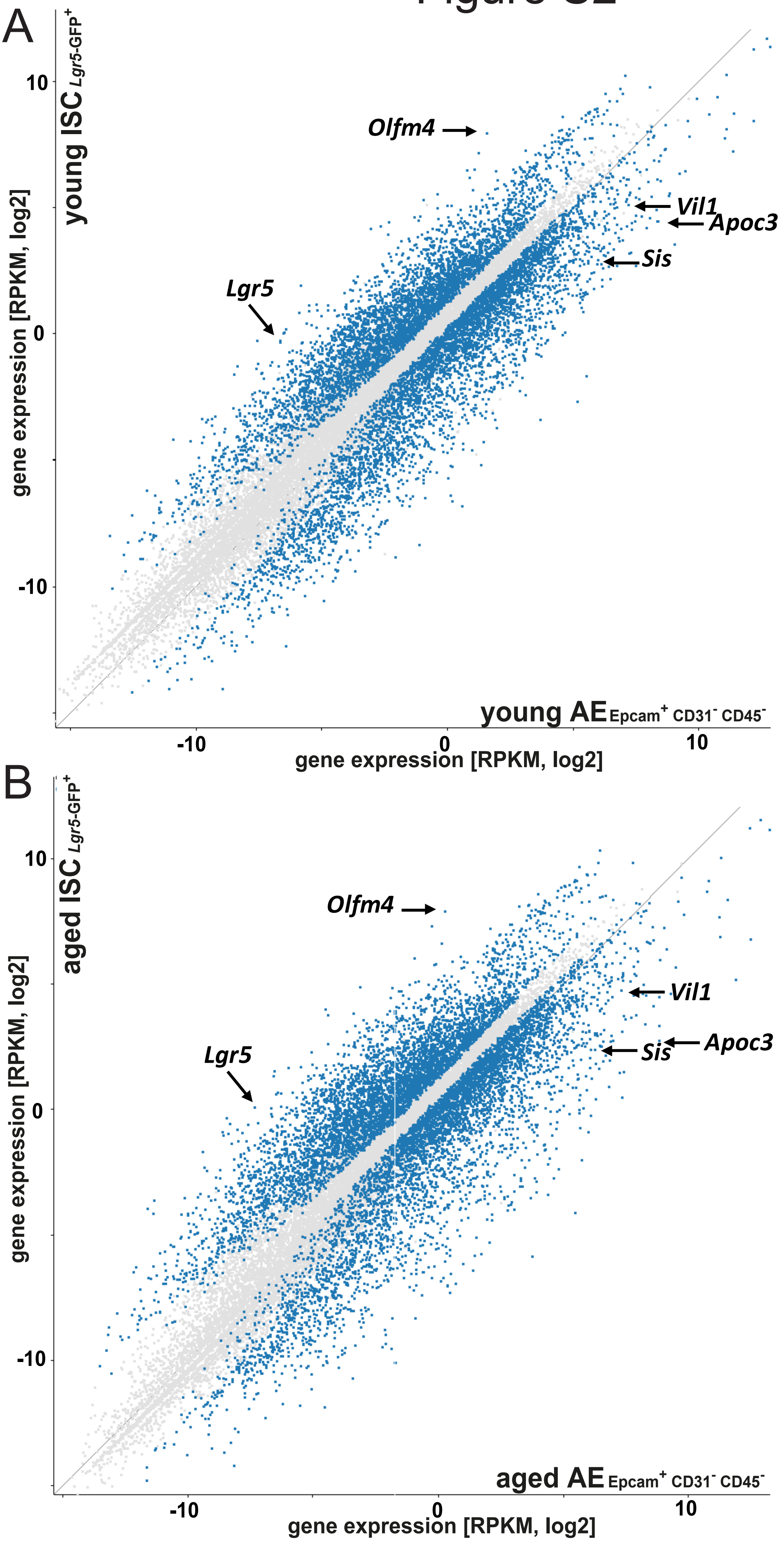
A
young ISC Lgr5-GFP+
gene expression [RPKM, log2]
young AE Epcam+ CD31- CD45-
gene expression [RPKM, log2]
Olfm4
Vil1
Apoc3
Sis
Lgr5
10
0
-10
-10
0
10
B
aged ISC Lgr5-GFP+
gene expression [RPKM, log2]
aged AE Epcam+ CD31- CD45-
gene expression [RPKM, log2]
Olfm4
Vil1
Apoc3
Sis
Lgr5
10
0
-10
-10
0
10

Figure S2. Expression of intestinal cell type specific epithelium markers during ageing. Scatterplots show relative expression levels as log2 scale RPKM (Reads Per Kilobase of transcript per Million mapped reads) quantitations in young (**A**) and aged (**B**) samples with genes significantly up/down-regulated between cell types of same age highlighted in blue (DESeq2 test with cut-off FDR<0.05, n=3). A subset of known ISC/AE cell type markers is labeled.

Figure S3

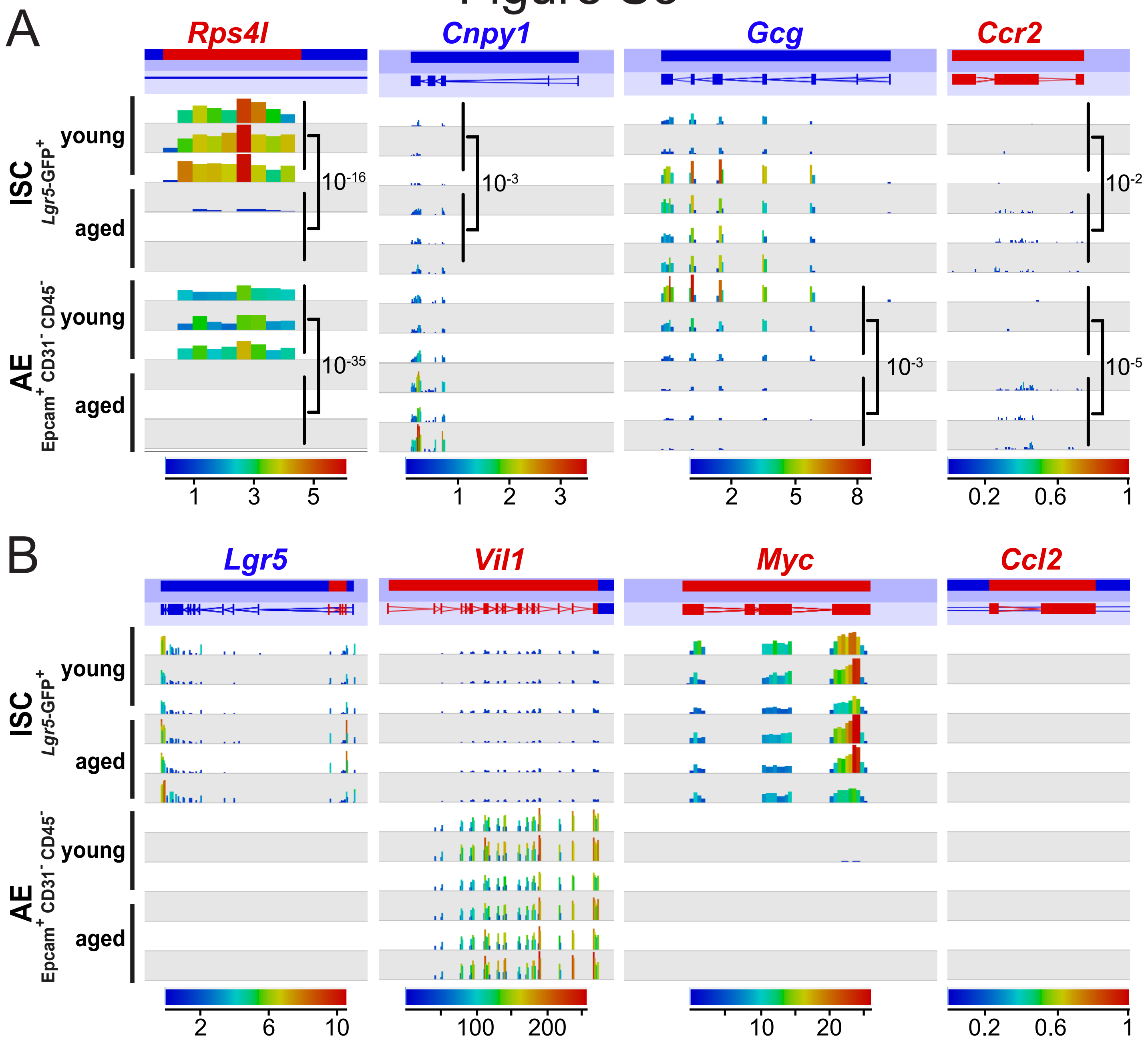
A
Rps4l
Cnpy1
Gcg
Ccr2
ISC
Lgr5-GFP+
young
aged
AE
Epcam+ CD31- CD45-
young
aged
10-16
10-3
10-2
10-35
10-3
10-5
1 3 5
1 2 3
2 5 8
0.2 0.6 1
B
Lgr5
Vil1
Myc
Ccl2
ISC
Lgr5-GFP+
young
aged
AE
Epcam+ CD31- CD45-
young
aged
2 6 10
100 200
10 20
0.2 0.6 1

Figure S3. Gene expression changes on ageing in the small intestinal epithelium. (**A**-**B**) RPKM-normalized RNA-seq track examples. 100 bp running window strand specific quantitation, linear scale. Significant aged/young changes in gene expression identified by DESeq2 are indicated with corresponding FDR values (cut-off FDR<0.05, n=3). (**A**) Genes differentially expressed between young and aged ISC and AE samples, as shown in Fig. 1E: *Rps4l* (0.94 kb), *Cnpy1* (45 kb), *Gcg* (9.1 kb) and *Ccr2* (12 kb). (**B**) Additional genes with no age-linked changes: *Lgr5* (137 kb), *Vil1* (26 kb), *Myc* (20 kb) and *Ccl2* (1.8 kb).

Figure S4

A

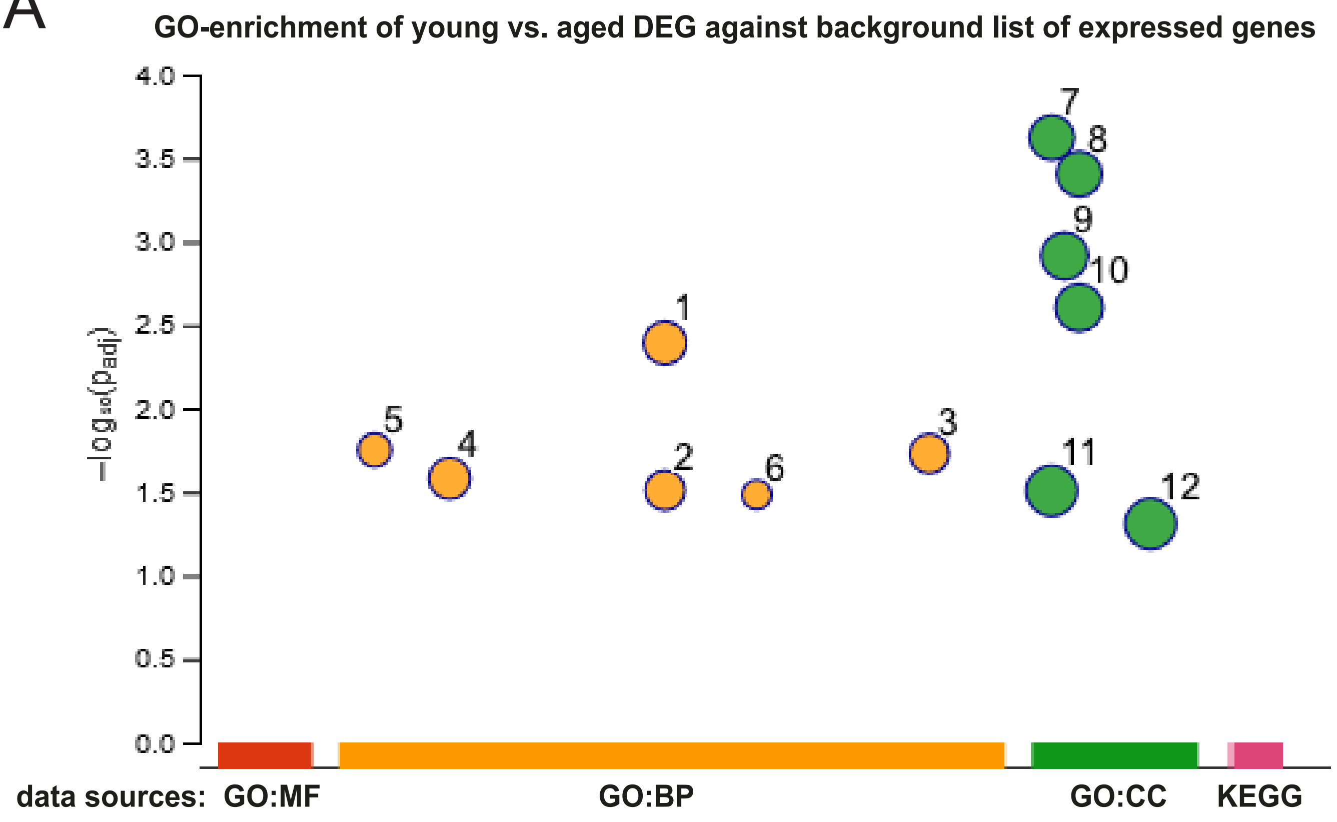

ID	source	term ID	term name	Padj
1	GO:BP	GO:0032101	regulation of response to external stimulus	4.053×10^{-3}
2	GO:BP	GO:0032102	negative regulation of response to external stimulus	3.092×10^{-2}
3	GO:BP	GO:0050727	regulation of inflammatory response	1.868×10^{-2}
4	GO:BP	GO:0006954	inflammatory response	2.623×10^{-2}
5	GO:BP	GO:0002275	myeloid cell activation involved in immune respon...	1.775×10^{-2}
6	GO:BP	GO:0036230	granulocyte activation	3.284×10^{-2}
7	GO:CC	GO:0005887	integral component of plasma membrane	2.393×10^{-4}
8	GO:CC	GO:0031226	intrinsic component of plasma membrane	3.942×10^{-4}
9	GO:CC	GO:0016021	integral component of membrane	1.222×10^{-3}
10	GO:CC	GO:0031224	intrinsic component of membrane	2.490×10^{-3}
11	GO:CC	GO:0005886	plasma membrane	3.111×10^{-2}
12	GO:CC	GO:0071944	cell periphery	4.905×10^{-2}

Figure S4. DEG aged vs. young GO-enrichment. Significantly enriched gene ontology annotations from the indicated databases (cut-off $P<0.05$, See Supplementary Table 1 for analysis parameters and detailed results). MF: Molecular Function, BP: Biological Process, CC: Cellular Component, KEGG: KEGG-Pathway.

Figure S5

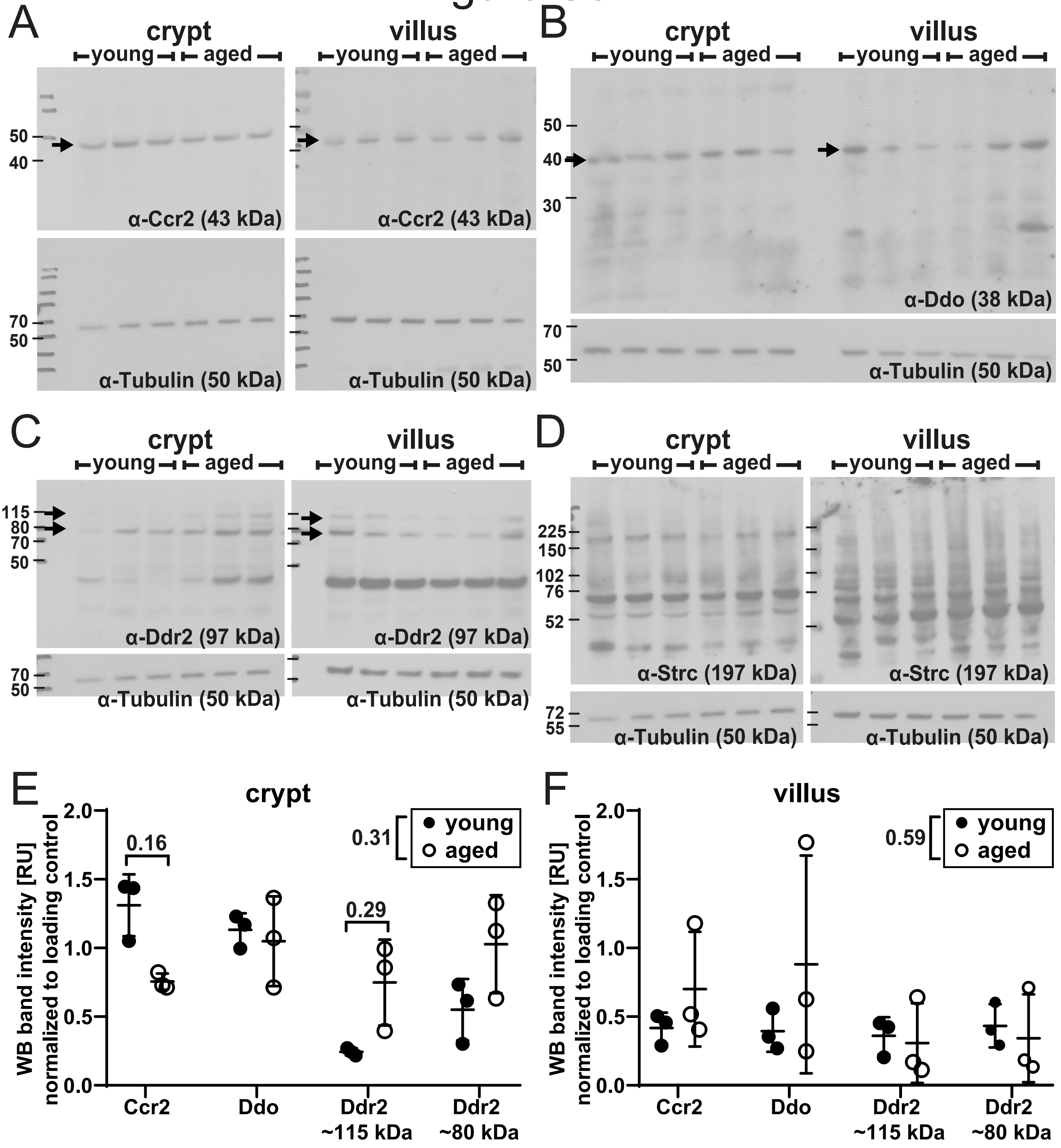

A
crypt
villus
young
aged
50
40
α-Ccr2 (43 kDa)
70
α-Tubulin (50 kDa)
B
30
α-Ddo (38 kDa)
C
115
80
α-Ddr2 (97 kDa)
D
225
150
102
76
52
72
55
α-Strc (197 kDa)
E
WB band intensity [RU]
normalized to loading control
0.16
0.31
0.29
2.0
1.5
1.0
0.5
0.0
Ccr2
Ddo
Ddr2
~115 kDa
~80 kDa
F
0.59

Figure S5. Protein levels of DEG during ageing. (**A**-**D**) Western blots of selected DEG-encoded proteins: Ccr2 (**A**), Ddo (**B**), Ddr2 (**C**) and Strc (**D**) with tubulin as loading control. Tubulin blots for crypts in panels A, C and D are the same. Tubulin blots for villi in panels A and D are the same. (**E**, **F**) Quantitation of arrow-indicated bands with normalization to loading control. SD indicated by error bars. No significant old/young changes in protein levels were identified by 2-Way ANOVA (cut-off $P<0.05$, $n=3$, selected P-values indicated. See also Supplementary Table 3).

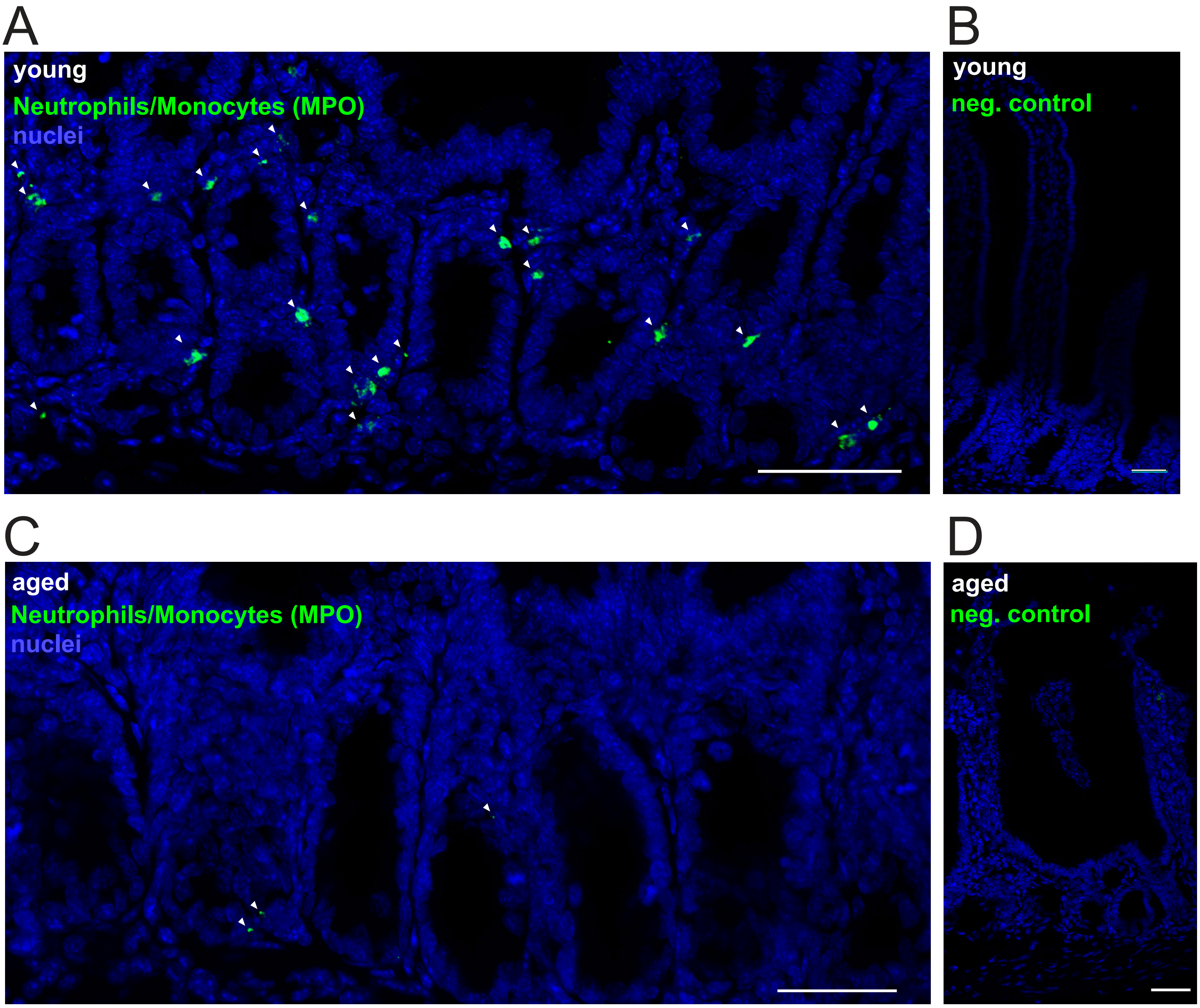
A
young
Neutrophils/Monocytes (MPO)
nuclei
B
young
neg. control
C
aged
Neutrophils/Monocytes (MPO)
nuclei
D
aged
neg. control

Figure S6. Neutrophils and monocytes during ageing. (**A**, **B**) Increased magnification of young (**A**) and aged (**B**) small intestinal crypt tissue in Fig. 3 A and B showing the sub-epithelial localization of Myeloperoxidase (MPO)-positive cells (green). Scored cells are indicated. Scale bars: 40 µm. (**C**, **D**) No-primary antibody control in young and aged SI.
